# *FAT1* expression in T-cell acute lymphoblastic leukemia (T-ALL) modulates proliferation and WNT signaling

**DOI:** 10.1038/s41598-023-27792-0

**Published:** 2023-01-18

**Authors:** Sven Liebig, Martin Neumann, Patricia Silva, Jutta Ortiz-Tanchez, Veronika Schulze, Konstandina Isaakidis, Cornelia Schlee, Michael P. Schroeder, Thomas Beder, Luc G. T. Morris, Timothy A. Chan, Lorenz Bastian, Thomas Burmeister, Stefan Schwartz, Nicola Gökbuget, Liliana H. Mochmann, Claudia D. Baldus

**Affiliations:** 1 Charité - Universitätsmedizin Berlin, corporate member of Freie Universität Berlin and Humboldt-Universität zu Berlin, Department of Hematology, Oncology and Cancer Immunology, Hindenburgdamm 30, 12203 Berlin, Germany; 2 German Cancer Research Center (DKFZ), Heidelberg, Germany; 3 German Cancer Consortium (DKTK), Heidelberg, Germany; 4 University Hospital Schleswig-Holstein, Campus Kiel, Department of Hematology and Oncology, Kiel, Germany; 5 Berlin Institute of Health at Charité - Universitätsmedizin Berlin, Core Facility Genomics, Berlin, Germany; 6 Center for Immunotherapy and Precision Immuno-Oncology, Cleveland Clinic, Cleveland, OH 44195 USA; 7 Immunogenomics and Precision Oncology Platform, Memorial Sloan Kettering Cancer Center, New York, NY 10064 USA; 8 Department of Medicine II, Hematology/Oncology, Goethe University Hospital, Frankfurt/Main, Germany; 9 Institute of Pathology, Ludwig-Maximilians-University Munich, Munich, Germany

**Keywords:** Cancer genetics, Cancer epigenetics, Acute lymphocytic leukaemia

## Abstract

FAT atypical cadherin 1 (FAT1), a transmembrane protein, is frequently mutated in various cancer types and has been described as context-dependent tumor suppressor or oncogene. The *FAT1* gene is mutated in 12–16% of T-cell acute leukemia (T-ALL) and aberrantly expressed in about 54% of T-ALL cases contrasted with absent expression in normal T-cells. Here, we characterized *FAT1* expression and profiled the methylation status from T-ALL patients. In our T-ALL cohort, 53% of patient samples were *FAT1* positive (FAT1pos) compared to only 16% *FAT1* positivity in early T-ALL patient samples. Aberrant expression of *FAT1* was strongly associated with *FAT1* promotor hypomethylation, yet a subset, mainly consisting of TLX1-driven T-ALL patient samples showed methylation-independent high *FAT1* expression. Genes correlating with *FAT1* expression revealed enrichment in WNT signaling genes representing the most enriched single pathway. *FAT1* knockdown or knockout led to impaired proliferation and downregulation of WNT pathway target genes (*CCND1*, *MYC*, *LEF1)*, while *FAT1* overexpressing conveyed a proliferative advantage. To conclude, we characterized a subtype pattern of *FAT1* gene expression in adult T-ALL patients correlating with promotor methylation status. *FAT1* dependent proliferation and WNT signaling discloses an impact on deeper understanding of T-ALL leukemogenesis as a fundament for prospective therapeutic strategies.

## Introduction

Human FAT atypical cadherin 1 (FAT1) is a transmembrane protocadherin, encoded by a gene localized at chr. 4q35.2 and highly conserved in its structure. It is a homologue of Drosophila tumor suppressor *fat*, known to be essential during developmental processes like cell polarity, proliferation and cell survival in drosophila and zebrafish^1–3^. In vertebrates, *FAT1* homologues are highly expressed in various fetal epithelia, yet lack of expression in mice was lethal and associated with brain and kidney defects^4,5^. In human, mutations or structural aberrations of the *FAT1* gene are associated with numerous developmental disorders like 4q-syndrome, nephropathy and syndactyly^1,6,7^, mental diseases such as bipolar or autism spectrum disorder^8–10^ or kidney diseases such as glomerulotubular nephropathy^11^.

*FAT1* mutations have been reported in various cancer types including glioblastoma, colorectal cancer and head and neck cancer^1,12^. In head and neck cancer, *FAT1* mutations have been described as marker for disease progression and adverse overall survival (OS) despite enrichment in cisplatin responders^13,14^. Therefore, *FAT1* mutated HPV-negative head and neck cancer is considered a unique subtype with respect for genetic landscape and prognosis^15–17^. Considering pathophysiology, FAT1 expression was correlated with hypomethylation of CpG islands in the *FAT1* gene^18^.

In hematological malignancies, *FAT1* mutations were detected in peripheral T-cell lymphoma. Here, the authors demonstrated *FAT1* mutations to be associated with inferior OS compared to wild-type^19^.

*FAT1*, originally cloned from the T-cell acute lymphoblastic leukemia (T-ALL) cell line Jurkat^4^, is mutated in 12–16% of T-ALL patients^20,21^. Furthermore, in a combined next-generation sequencing approach analyzing 121 B- and T-ALL patients, *FAT1* was the gene most frequently mutated^22^. Regarding expression, we and others have reported aberrant *FAT1* mRNA expression in T-ALL, yet *FAT1* was not found to be expressed in hematopoietic progenitor cells, unselected bone marrow or peripheral blood from healthy donors^20,23^. Aberrant *FAT1* expression occurred in 54% of T-ALL patients without significant correlation to its mutational status^20,21^. In addition, a truncated *FAT1* isoform lacking exons 1 to 24 and labelled as ΔFAT1 (Ensembl FAT1-004 transcript, EST transcript BX362336.2) was described in T-ALL^24^.

OS was inferior, although not statistically significant, for FAT1 positive (FAT1pos) T-ALL patients^20^, whereas in pediatric B-cell lymphoblastic leukemia (B-ALL), *FAT1* expression was associated with an impaired OS and higher probability of relapse^23^.

The molecular and biochemical background of *FAT1* mutations, aberrant expression and regulation in cancer is poorly understood. Treatment of cancer cell lines with hypomethylating agents induced *FAT1* expression^25^ which suggests, that oncogenic promotor hypomethylation might explain dysregulated *FAT1* expression. Concerning biological functions, Morris et al. have shown a tumor suppressive role of *FAT1* by WNT pathway inhibition controlling cancer cell growth, cell cycling, and size independent cell–cell adhesion in glioma, immortalized human astrocytes and xenograft models as an explanation for impaired OS in *FAT1*-mutated patients with glioblastoma^12^. Likewise, FAT1 binding β-Catenin inhibits proliferation and metastasis of cervical cancer cells^26^. Regarding the FAT1-WNT pathway interaction in T-ALL, we and others have reported the importance of dysregulated WNT signaling in leukemogenesis and especially in T-ALL^27–29^.

In our study we characterize *FAT1* expression in adult T-ALL by combining RNA-sequencing (RNA-seq) expression and methylome data of a large patient cohort. To better understand the functional role of *FAT1* in T-ALL, we applied gene set enrichment (GSEA) as well as pathway enrichment analyses from T-ALL patient data and characterized FAT1 overexpression (OE), knockdown (KD) and knockout (KO) regarding a proliferative effect and FAT1-WNT pathway interaction in T-ALL.

## Results

### Aberrant FAT1 expression in adult T-ALL

**Figure 1:**
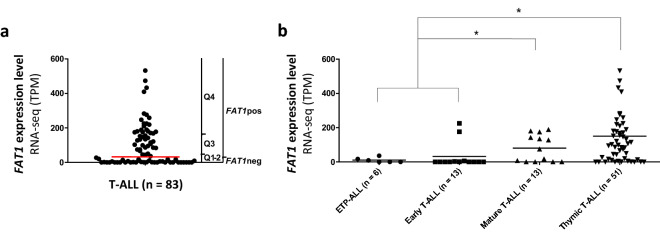
Aberrant *FAT1* expression in T-ALL (**a**) FAT1 expression data analyzed by RNA-seq (HighSeq 2000, 125 bp, ~ 30 million reads/sample) was available for n = 83 diagnostic T-ALL samples from adult patients (median age 32 years, range 17–59 years; including n = 19 early T-ALL and ETP-ALL, n = 51 thymic T-ALL and n = 13 mature T-ALL. *FAT1* positivity was considered by a cutoff at Transcripts Per Million (TPM) 30 defining those two groups with high (FAT1pos, n = 45, median TPM 155, range 36–1368) or very low/negative *FAT1* expression (FAT1neg, n = 38, median TPM 0.5, range 0–27). T-ALL patients were subdivided into quartiles according to expression. Q1-Q2 represented *FAT1* negative or *FAT1* very low and Q3-Q4 *FAT1* high expression. One outlier (TPM 1368) was removed from the diagram but included in statistical analyses. (**b**) *FAT1* expression varied between phenotypical T-ALL subgroups. *FAT1* expression was significantly higher in mature (54% FAT1pos) and thymic T-ALL (69% FAT1pos) compared to early-/ETP-ALL. (16% FAT1pos; *p < 0.05).

Investigating RNA-seq based transcriptome data from a T-ALL cohort of n = 83 adult patients we confirmed *FAT1* overexpression in 53% of T-ALLs (FAT1pos, n = 45, median TPM 155, range 36–1368; Fig. 1a), whereas 47% of patients had very low/negative *FAT1* expression (FAT1neg, n = 38, median TPM 0.5, range 0–27; Fig. 1a). Remarkably, *FAT1* expression varied across immunophenotypic T-ALL subtypes. In fact, 69% of thymic T-ALL were FAT1pos compared to 54% in mature (p = 0.25, Fig. 1b) and only 16% in ETP-/early-T-ALL (p = 0.02, Fig. 1b). Accordingly, for FAT1pos patients immunophenotypic expression levels of CD1a (p = 0.005), CD4 (p < 0.0001), and CD8 (p = 0.001) were significantly higher compared to cells from FAT1neg patients, which disclosed a more frequent coexpression of the early myeloid antigens CD13 (p = 0.002) and CD33 (p = 0.008) (Supplementary Fig. 1a,b). Interestingly, these differences in maturity markers between FAT1pos and FAT1neg were also apparent within the T-ALL immunophenotypic subgroups (Supplementary Fig. 1c–e). Investigating the independent T-ALL RNA-Seq based dataset from Liu et al.^30^, we could validate our findings regarding the correlation between FAT1 expression and the immune phenotypes reflecting maturation stages of T-ALL. In the n = 264 samples from pediatric and young adult T-ALL patients (reported by Liu et al.^30^) we found FAT1 positivity (FAT1pos) in 53% of cortical T-ALL (p < 0.0001; Supplementary Fig. 2a) and 44% of post-cortical T-ALL (p = 0.01; Supplementary Fig. 2a) compared to only 17% positivity in pre-cortical T-ALL samples. Notably, n = 41 samples were not annotated for the T-ALL immune phenotype here. Levels for CD4- and CD8 expression were also higher with lower CD33 expression in this dataset (Supplementary Fig. 2b–d). However, cortical T-ALL marker CD1a was not significantly upregulated for FAT1 pos T-ALL in this validation dataset (p = 0.07; Supplementary Fig. 2e).

In this comprehensive T-ALL dataset, we also determined *FAT1* expression according to molecular T-ALL subtypes. Here, *FAT1* was highly expressed in the majority of TAL1/2, TLX1 and LMO1/2 samples, but not in those with a HOXA, TLX3, LYL1 and NKX2-1 molecular subtype (Supplement Fig. 3).

### Promotor hypomethylation mediates aberrant *FAT1* expression in T-ALL

**Figure 2:**
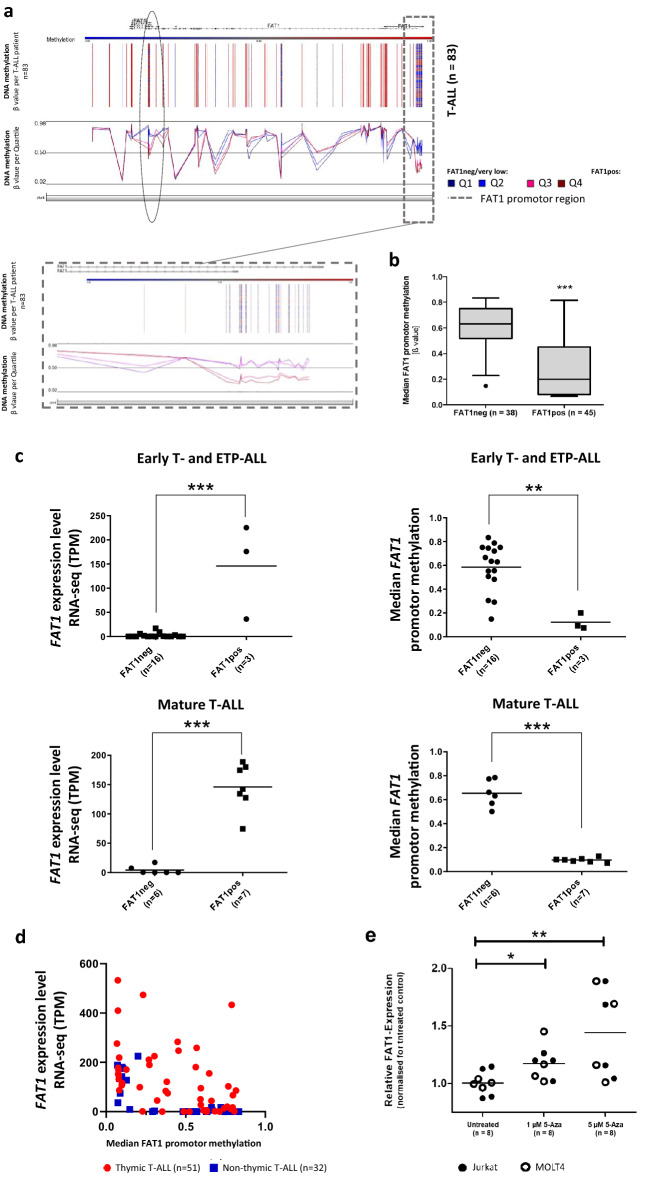
Promotor hypomethylation and *FAT1* expression in T-ALL. (**a**) Patient samples were assessed for DNA methylation by HumanMethylation450 BeadChip. β values for DNA methylation for each patient (upper plot) and mean DNA methylation according to expression clusters Q1-Q4 (lower plot) are depicted for chr 4q34-35. Q1/Q2 mainly represent FAT1neg T-ALL patients. The promotor region is marked by the dotted box. (**b**) Tukey boxplot of median promotor methylation comparing FAT1pos (mean methylation score: 0.28; n = 38) and FAT1neg (mean methylation score: 0.61; n = 45) T-ALL samples. Median promotor methylation was significantly higher (p < 0.001) in FAT1neg T-ALL samples. (**c**) Inverse correlation between high *FAT1* expression and FAT1 promotor hypomethalation was particularly significant for mature T-ALL compared to ETP/early T-ALL. (**d**) A correlation plot between *FAT1* expression and median promotor methylation with hyperbolic correlation (R^2^ = 0.63) is presented in non-thymic T-ALL samples. No such clear correlation could be shown in thymic T-ALL. (**e**) Treatment with demethylating agent 5-Azacytidine (5-Aza) for 24 h in T-ALL cell lines (Jurkat and Molt-4) led to a dose-dependent *FAT1* upregulation (*p < 0.05; **p < 0.01; ***p < 0.01).

Exploring the regulation of *FAT1* expression, we combined transcriptomic and methylome data within the same T-ALL patient cohort. Methylation of CpGs within the *FAT1* promotor were significantly higher between expression Quartiles Q1–2 compared to Q3–4 (mean of median CpG methylation 0.62 vs. 0.33, p = 0.0002; Fig. 2a).

A second site of the *FAT1* transcript (ENSG00000083857) with differently methylation was identified near to the 3′ end (marked with ellipse in Fig. 2a). The 3′ methylation site described here is located directly upfront a truncated FAT1 isoform, likely ΔFAT1 (Ensembl FAT1-004 transcript, EST transcript BX362336.2; Supplementary Fig. 7c)^24^.

Moreover, we compared *FAT1* DNA methylation and *FAT1* expression for the phenotypic subtypes ETP/ early, thymic and mature T-ALL. Among all non-thymic T-ALL samples, a strong negative correlation between *FAT1* expression and promotor methylation was observed (Fig. 2a–c). Median FAT1 promotor methylation was significantly higher in FAT1pos compared to FAT1neg samples (p < 0.001; Fig. 2b) with a Pearson correlation coefficient r = −0.3397 [95% CI − 0.5176 to − 0.1338] reflecting a significant correlation with p = 0.007. For the non-thymic T-ALL samples, this correlation was even more striking (R^2^ = 0.6; Pearson r = −0.7546; 95% CI [− 0.8735 to − 0.5507] and p < 0.0001; Fig. 2c,d).

Correlation analyses in thymic T-ALL were less consistent compared to the black-and-white pattern in non-thymic T-ALL (Fig. 2c). Overall, a strong correlation between *FAT1* gene expression and *FAT1* promotor hypomethylation was also present in thymic T-ALL (Supplementary Fig. 4a,b). However, a considerable subgroup of thymic T-ALL patients showed no correlation between *FAT1* expression and promotor hypomethylation (Supplementary Fig. 4a,b). This particular subgroup had predominantly *TLX1* oncogenic expression as molecular characteristic (Supplementary Fig. 4c). In addition, these patients had significantly higher levels of CD1a as assessed by flow cytometry (Supplementary Fig. 4d).

To further study epigenetic regulation of *FAT1* expression we treated T-ALL cell lines Jurkat and Molt-4 with the hypomethylating agent 5-Azacytidine at 1 and 5 µM for 24 h in vitro and analyzed *FAT1* mRNA expression by Real-Time PCR (RT-PCR), discovering a dose-dependent *FAT1* upregulation (FC = 1.45; p = 0.008 for 5 µM; Fig. 2e).

### *FAT1* is correlated with maturity and distinct pathway patterns in T-ALL

**Figure 3:**
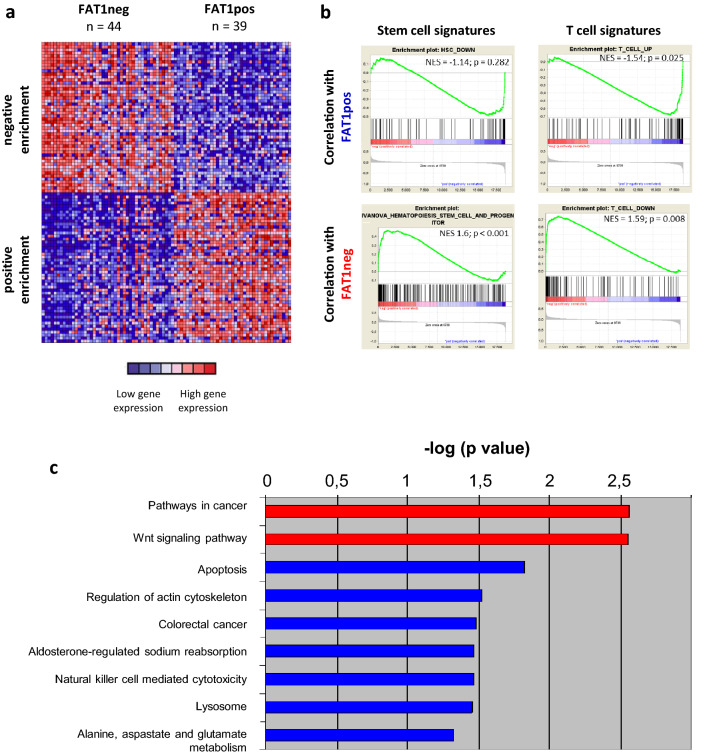
*FAT1*-dependent gene expression in T-ALL (**a**) Heatmap of coexpressed top 50 most up- and downregulated genes according to expression in *FAT1* positive or negative samples is depicted for a dataset of n = 83 T-ALL patients. FAT1pos samples display a *FAT1*-dependent expression pattern. (**b**) GSEA comparing FAT1pos with FAT1neg patients showed a strong correlation between *FAT1* expression and T-ALL maturity with respect for mature T-cell and stem cell signatures. (**c**) Modules *Pathways in cancer* and *WNT signaling* are significantly upregulated and most prominent in FAT1pos thymic T-ALL patient samples.

Next, we investigated the transcriptional program in an available microarray expression data set of n = 83 T-ALL patients (characterized in^31–33^). The analysis of this available microarray expression data set revealed 39 of 83 patients as FAT1pos, while the remaining 44 patients were classified as FAT1neg. Mapping most coregulated genes according to *FAT1* expression using GSEA revealed a *FAT1*-dependent gene expression signature (Fig. 3a). Genes correlated to the FAT1pos genotype with highest enrichment scores included *PBK*, *MAL*, *TLX1*, *PRKCA* and *RAG1* (Supplementary Tables 1,3,4).

We found upregulation of genes associated with T-cell differentiation for FAT1pos (NES 1.54, p = 0.025, FDR q = 0.056; Fig. 3b, Supplementary Tables 5,6) and of the stem cell signature published by Ivanova et al.^34^ for FAT1neg patients (NES 1.6, p < 0.0001, FDR q = 0.06; Fig. 3b, Supplementary Tables 5,7). Accordingly, genes typically downregulated in mature T-cells were highly enriched in FAT1neg T-ALL samples (NES 1.59, p = 0.008, FDR q = 0.052; Fig. 3b, Supplementary Tables 5,8). We validated our findings performing GSEA with T-cell differentiation and stem cell signatures in the dataset from Liu et al.^30^ (Supplementary Fig. 5a,b).

This held true in our data for thymic T-ALL as the prognostic most favorable subgroup (n = 36, 72% FAT1pos vs. 28% FAT1neg). Here, a total count of 691 genes was represented by annotated probe sets significantly coregulated with *FAT1* expression (Supplementary Table 3). Significantly enriched pathways are depicted as − log(p) in Fig. 3c pointing at the module “WNT signaling pathway” regarding significance.

### FAT1 is a modulator of proliferation and WNT signaling in T-ALL

**Figure 4:**
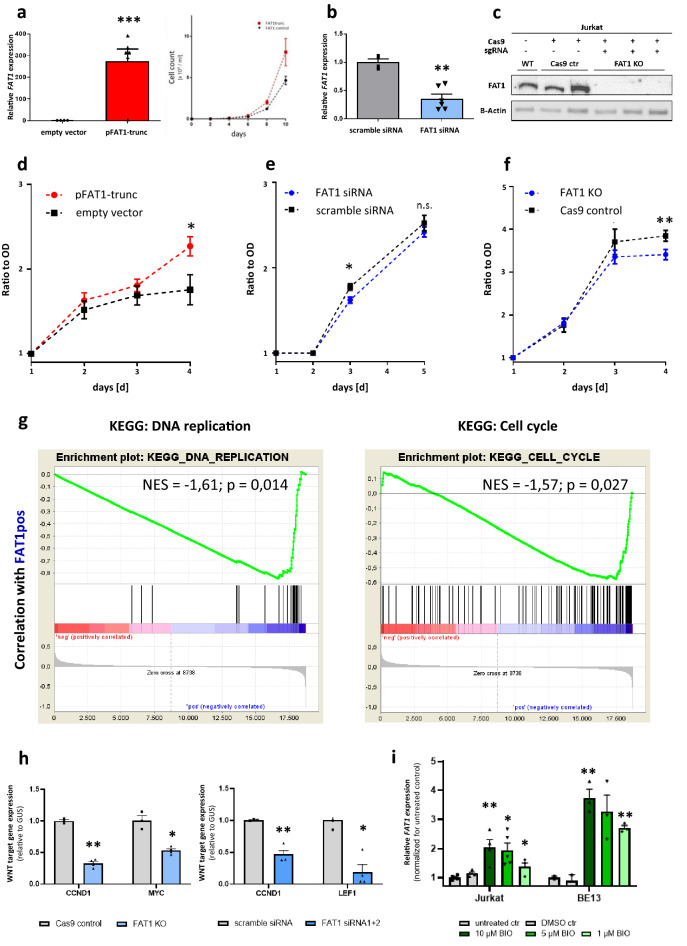
*FAT1* induces proliferation in T-ALL and interacts with WNT signaling (**a**) *FAT1* overexpression (OE) using a truncated *FAT1* plasmid (pFAT1-trunc) in Jurkat demonstrated by RT-PCR (basic and transgenic expression). FAT1 overexpression led also to higher cell proliferation. Cells were counted every second day using a conventional trypan-blue staining comparing FAT1trunc and FAT1 control cells. (**b**) Specific siRNA *FAT1* KD in Jurkat with two independent siRNAs assessed by RT-PCR. (**c**) CRISPR/Cas9-mediated *FAT1* Knockout (KO) in T-ALL cell line Jurkat validated by Western blot analysis (full-length gel provided as supplementary information file). (**d**–**f**) WST-1 proliferation assay after (**d**) *FAT1* OE, (**e**) KD or (**f**) KO proofed a proliferative advantage of high *FAT1* expression in Jurkat as a T-ALL model. Data plotted as ratio to optical density (OD) after normalization at days [d] 1, 2, 3 and 4 or 5. (**g**) GSEA for *KEGG DNA REPLICATION* and *KEGG CELL CYCLE* were performed analyzing all FAT1pos and FAT1neg T-ALL samples from the patient cohort. (**h**) Representative results showing downregulation of WNT target genes, namely *CCND1*, *LEF1* and *MYC* due to *FAT1* KO (CCND1: FC = −3.0, p = 0.002; MYC: FC = −1.86, p = 0.026) or FAT1 KD (CCND1: FC = −2.13, p = 0.004; LEF1: FC = −5.26, p = 0.016) as analyzed by RT-PCR. (**i**) Dose-dependent upregulation of FAT1 expression caused by treatment with WNT pathway activator BIO in the T-ALL cell line Jurkat and BE13 underlines FAT1-WNT pathway interaction (n.s.: not significant; *p < 0.05; **p < 0.01).

Driven by transcriptomic data, functional consequences of aberrant *FAT1* expression with respect for proliferation and WNT signaling were studied in vitro. We established *FAT1* OE, *FAT1* KD and *FAT1* KO (Fig. 4a–c) and performed WST-1 proliferation assays in at least two independent and representative experiments. Jurkat T-ALL cells transfected with a truncated but functional *FAT1* plasmid (pFAT1-trunc, characterized by^12^) showed increased proliferation compared to the control empty vector pcDNA3.1 (p = 0.005; Fig. 4d). The observation of cell proliferation over a prolonged time period of 10 days led to enriched counts for FAT1high Jurkat T-ALL cells further underlining the proliferative advantage of high *FAT1* expression as shown by WST proliferation assays shown (Fig. 4a; d–f).

Respectively, *FAT1* KD (Relative increase: 0.12; p = 0.017 ad day 3, no significant difference at day 5, Fig. 4e) and *FAT1* KO (Relative increase: 0.44; p = 0.002 at day 4; Fig. 4f) resulted in an impaired cell proliferation also indicating a dependence on *FAT1* expression levels. We recapitulated the association between FAT1 expression and proliferation in a genome-wide approach using GSEA. Enrichment analyses revealed a strong enrichment for genes of KEGG modules “DNA replication” and “cell cycle” for the FAT1-positive phenotype (Fig. 4g, KEGG DNA replication: NES 1.61, p = 0.014, KEGG cell cycle: NES 1.57, p = 0.027).

Considering *FAT1* WNT pathway interaction, GSEA from the comprehensive Liu et al. dataset^30^ showed enrichment for the WNT pathway module in the FAT1 positive subgroup (Supplementary Fig. 6a). We then investigated WNT pathway target gene expression by RT-PCR. Results were heterogeneous reflecting complexity of biochemical signaling, but a strong downregulation for WNT target genes *CCND1* and *MYC* (p = 0.002/p = 0.026, Fig. 4g) after *FAT1* KO or *CCND1* and *LEF1* (p = 0.004/p = 0.016, Fig. 4h) after *FAT1* KD was noticeable. We further treated different T-ALL cell lines such as Jurkat with WNT pathways activators and saw strongest effects 6-Bromoindirubin-3′-oxime (BIO). T-ALL cell lines Jurkat and BE13 were treated with BIO and *FAT1* mRNA expression was assessed after 24 h. BIO did not induce significant growth inhibition or apoptosis but dose-dependent *FAT1* upregulation with 2.05-fold increase for 10 µM in Jurkat (p = 0.008; Fig. 4i) and 3.8-fold increase for 10 µM BIO in BE13 (p = 0.003; Fig. 4i). Notably, WNT pathway inhibition with compound XAV-939 led to dose-dependent downregulation of FAT1 (Supplementary Fig. 6b).

## Discussion

Acute lymphoblastic leukemia, a disease driven by neoplastic proliferation as a result of malignant transformation of lymphoid progenitor cells, represents about 20% of adult leukemia^35,36^. In contrast to B-lineage ALL, T-ALL presents about 25% of ALL cases and, in case of all non-thymic T-ALL subtypes, is an independent risk factor for adverse overall survival, especially when relapsed after first induction therapy^37,38^. Unfortunately, stagnant progress has been achieved for new therapy approaches in T-ALL in addition or as an alternative to classical chemotherapy. Novel molecular targets are required to develop individualized and targeted concepts, as based on antibody, small molecule or CAR-T strategies in T-ALL. An interesting candidate is *FAT1*, exclusively expressed in hematopoietic malignancies like AML or ALL but not physiologically in normal hematopoiesis^20^.

The leukemia specific expression of *FAT1* in T-ALL leukemogenesis highlights its potential impact for translation into diagnostics and therapeutics. With respect to clinical diagnostic application, *FAT1* has already been proposed as MRD marker in the context of B-ALL^39^. Considering first therapeutic approaches, a FAT1-derived epitope was successfully tested as anti-CRC cancer vaccine in a murine model and therapeutic antibodies or antibody-conjugated drugs directed against *FAT1* for CRC are under development, mechanistically applicable also for T-ALL^40–42^.

FAT1 is among the most frequently mutated genes in T-ALL^20–22^ and was confirmed to be aberrantly expressed in 54% of T-ALL patients. In a previously published study we have described an association of *FAT1* expression and T-ALL maturation stages as well as a negative correlation with stem cell genes *MN1*, *BAALC* and *IGFBP7*^20^. Here, we broadened insights in FAT1 expression in T-ALL. We could show an enrichment of stem cell signatures in GSEA in FAT1 negative and vice versa an enrichment for T-cell signatures in FAT1 positive patient samples. Furthermore, single stem cell-associated genes of this genesets were also significantly downregulated within each phenotypical T-ALL subtype. Importantly, maturation markers based on flow cytometry were also enriched within phenotypical subtypes according to FAT1 positivity. Taken together, we show not only a correlation between maturation and *FAT1* expression on the level of candidate gene expression, but also with transcriptional programs and methylation profiles. It has to be discussed, whether *FAT1* might add valuable information to cytometry based phenotypic classification in T-ALL.

Knowledge about regulation of aberrant *FAT1* expression in cancer is limited. As *FAT1* mutations and expression in T-ALL do not necessarily correlate, other mechanisms of regulation likely exist^20^. Promotor hypomethylation was hypothesized as an explanation for aberrant *FAT1* expression in HCC as treatment with hypomethylating agents 5-Aza-2′deoxacytidine, adenosine-2′,3′-dialdehyde or S-adenosyl-L-methionine augmented *FAT1* expression in HCC cell lines^25^.

Here, we correlated aberrant *FAT1* expression in T-ALL with promotor hypomethylation and discovered increased *FAT1* expression by drug treatment with the hypomethylating agents Decitabine or 5-Azacytidine. However, we did not observe a similar correlation with part of the thymic T-ALL subgroup suggesting other mechanisms, which could contribute to aberrant FAT1 expression in a complex biochemical regulation network. In fact, we could identify a *TLX1/HOX11* driven oncogenic background for the majority of methylation-independent FAT1 upregulated samples. Those patients had significantly higher levels of CD1a cell surface expression reflecting TLX1 related high CD1a expression and disruption of differentiation at the level of CD1a+ CD4+ and CD8+ early cortical thymocytes. The functional link between *FAT1* and *TLX1* is underlined by ChIP-seq profiling in T-ALL identifying FAT1 among the most prominent binding partners of TLX1^43^. Likewise, exceptional high *FAT1* expression levels could also be found in t(1;19)(E2A-PBX1) translocated BCP-ALL patients^23^. Taken together, regulation of *FAT1* expression is complex and further evaluation will be necessary to decipher precise mechanisms contributing to aberrant *FAT1* expression.

Notably, we identified a second site with significant differential methylation of the FAT1 locus (NM_005245) near to the 3′ end. This would support the findings from de Bock et al. regarding an epigenetic regulation site upfront of the truncated variant ΔFAT1 in T-ALL^24^.

Exploring *FAT1*-associated gene expression we concentrated on the most comprehensive subgroup of thymic T-ALL patients and found the WNT pathway to be the most enriched distinct pathway, which correlated with *FAT1* expression. Notably, a *FAT1*-WNT pathway association has already been identified in glioma and ovarian cancer patient samples and has been functionally shown in glioma cell lines^12^. Loss of FAT1 binding capacity for the key classical WNT pathway protein β-Catenin was caused by loss of *FAT1* expression in cancer and therefore resulted in β-Catenin translocation to the nucleus and downstream expression effects such as regulation of target genes *MYC* and *Cyclin D1*. Hence, FAT1 was proven to be a negative regulator of WNT signaling in glioma, consistent with a tumor suppressive character^12^. We revealed significant effects on identical WNT target genes upon *FAT1* OE, KD or KO. In contrast to findings in glioma, *FAT1* expression in T-ALL was positively linked to WNT target gene expression not fitting the model of a negative β-Catenin regulator. Likely, this fundamental difference is caused by an unidentified context-specific regulatory pathway network. Furthermore, WNT pathway activation by pathway activator BIO caused a *FAT1* upregulation whereas pathway inhibition by XAV-939 resulted in the opposite in preliminary experiments. This putative feedback mechanism has not been reported yet, but is likely to be regulated via *TCF/LEF*-binding sites within the FAT1 promoter region as forecasted by in silico means^44^. Concerning the TLX1-driven thymic T-ALL subgroup, others have reported the TLX1 mediated modulation of WNT signaling in T-ALL preventing thymocyte progression during differentiation^45–47^. The landscape of deregulated WNT signaling in T-ALL^27–29,48^ could thus be complemented by WNT pathway modulation upon *FAT1* aberrant gene expression.

Finally, positive regulation of WNT signaling by FAT1 and an enrichment of DNA replication and cell cycle suggests, that FAT1 might control proliferation in T-ALL. For *FAT1* expressing HCC Valetta et al. reported impaired proliferation due to *FAT1* suppression by short hairpin RNA^25^. Indeed, we found enriched cell proliferation in *FAT1* OE cells but decreased proliferation in *FAT1* KD or KO cells. Furthermore, a study published by de Bock et al. demonstrated the expression of a unique truncated FAT1 isoform in T-ALL, for which OE also resulted in an increased cell proliferation in T-ALL cell lines and collaborated with mutated *NOTCH1* as key driver in a majority of T-ALL cases^24^.

To summarize, this study contributes to a better understanding for the functional role of *FAT1* in T-ALL and deepens the knowledge of leukemogenesis by dissecting mechanisms leading to *FAT1* expression, FAT1-dependent proliferation and WNT pathway dysregulation.

## Methods

### Patient samples, expression and methylation data

Gene expression data analyzed by RNA-sequencing (RNA-seq) (HighSeq 2000, 100/125 bp Paired-end sequencing, ~ 30 million reads/sample) were available for n = 83 diagnostic T-ALL samples from adult patients (median age 32 years, range 17–59 years; including n = 19 early T-ALL and ETP-ALL, n = 51 thymic T-ALL and n = 13 mature T-ALL; Supplementary Fig. 1a,b) from the GMALL 07/2003 study cohort^49^. FAT1 positivity was considered by a cutoff at Transcripts Per Million (TPM) 30 defining those two groups with high (FAT1pos, n = 45, median TPM 155, range 36–1368, Fig. 1a) or very low/negative FAT1 expression (FAT1neg, n = 38, median TPM 0.5, range 0–27, Fig. 1a). For further analyses, patients were subdivided into *FAT1* expression quartiles (Q1–Q4, each quartile representing 25% of patients) with Q1-Q2 comprising predominantly FAT1neg patients. Phenotypic T-ALL stratification (early/immature ALL: CD2-, surface CD3-; thymic T-ALL: CD1a+; mature T-ALL: CD2+, surface CD3+/−) was set according to flow cytometry based immunophenotyping at the German Multicenter Study Group on Adult Acute Lymphoblastic Leukemia (GMALL) reference laboratory^50^. From this dataset DNA methylation data assessed by Infinium® HumanMethylation450 BeadChip (Illumina, San Diego, USA) were also available. The *FAT1* locus (NM_005245) was represented by 123 CpG sites. Methylation data are expressed as β values ranging from 0 to 1, which had been transformed according to signal intensity for methylated and unmethylated cytosine nucleotides. Investigation of methylation data was carried out as previously described^31,49^. To assess promotor methylation status, we calculated median values of the CpG sites within the FAT1 promotor region and compared median promotor methylation with RNA-seq based *FAT1* gene expression. Exploring *FAT1-*dependent gene expression in T-ALL we performed GSEA and Pathway enrichment analyses investigating expression data based on Affymetrix HG-U133 Plus 2.0 from another independent T-ALL cohort (n = 83 T-ALL patients) which has already been published (Geo Accession number GSE78132)^31–33^. Data were analyzed using Partek Genomics Suite 6.6 software (Partek Ink., St. Louis, Missouri, USA).

### Gene set enrichment and pathway enrichment analyses

For Gene set enrichment analyses (GSEA; GSEA software: Broad Institute, Inc., Massachusetts Institute of Technology, and Regents of the University of California) of T-cell maturity profiles comparing FAT1pos and FAT1neg gene expression profiles (GEP) for genes up- or downregulated in T-cells (“T_CELL_UP”, “T_CELL_DOWN”) or downregulated in hematopoietic stem cells (“HSC_DOWN”) were adopted from a previously published study from our group based on GEPs of T-ALL and hematopoietic differentiation stages defined as previously described (Additional File 2; Supplementary Table 5)^31,51^. GEP for genes upregulated in hematopoietic stem cells (HSC) was considered as previously described by Ivanova et al. (Additional File 2; Supplementary Table 5)^34^. GSEAs were realized with the GSEA software, desktop application version 4.0.1, from the Broad Institute (http://www.broadinstitute.org/gsea) and Molecular Signature Database (MSigDB)^52^. KEGG pathway modules “KEGG_CELL_CYCLE” and “KEGG_DNA_REPLICATION” for GSEA were taken from the MSigDB. For pathway enrichment analyses, we created lists of up- and downregulated genes comparing FAT1pos and FAT1neg T-ALL patient samples. As cutoff for differential expression, statistical significance with p ≤ 0.05 in ANOVA testing was considered resulting in a list of 691 differentially expressed genes for thymic T-ALL (Additional File 2; Supplementary Table 3). For pathway enrichment analysis of this gene list, the KEGG pathway annotation tool from the DAVID bioinformatics server (https://david.ncifcrf.gov/) was used^53,54^.

### Cell culture and drug treatment

Human T-ALL cell lines Jurkat (ACC-282) and Molt-4 (ACC-362), obtained from the German Resource Center for Biological Material (Braunschweig, Germany), were cultured in RPMI-1640 medium (Gibco, Thermo Fisher Scientific, Waltham, Massachusetts, USA). 5-Azacytidine (5-Aza) was purchased from Sigma-Aldrich, now a Merck company (Darmstadt, Germany).

### RNA isolation and Real-Time PCR

RNA was isolated from up to 5 × 10^6^ cells with the RNeasy Kit (Qiagen, Venlo, The Netherlands). For transcription from RNA to cDNA we used MMLV reverse transcriptase (Epicentre, Madison, USA). Measurement of FAT1 expression was done by Real-Time PCR (RT-PCR) using *FAT1* primers FAT1-forward: 5′-TGATCCCTGTCTTTCCAAGAAGCCT and FAT1-reverse: 5′-CGGCAGAG-GAACGCTTGGCA as well as the corresponding *FAT1* TaqMan probe: 5′-FAM-AGCCTTCCCAGCCATACAGTGCCCGGG-BHQ1 as previously described^55^. Expression of the house keeping gene β-Glucoronidase (*GUS*) served as internal control with primers GUS-forward: 5′-GAAAATATGTGGTTGGAGAGCTCATT, GUS-reverse: 5′-CCGAGTGAAGATCCCCTTTTTA and a TaqMan *GUS* probe: 5′-JOE-CCAGCACTCTCGTCGGTGACTGTTCA-BHQ1. For WNT target genes we performed a classical SYBR Green PCR assay as described by the manufacturer (SYBR® GreenER™ qPCR SuperMix, Thermo Fisher Scientific, Waltham, Massachusetts, USA). Sequences for WNT target gene primer pairs are:

**Table:** 

LEF1	LEF1_FWD	AATGAGAGCGAATGTCGTTGC
	LEF1_REV	GCTGTCTTTCTTTCCGTGCTA
CCND1	CCND1_FWD	GTGCTGCGAAGTGGAAACC
	CCND1_REV	ATCCAGGTGGCGACGATCT
MYC	MYC_FWD	GTCAAGAGGCGAACACACAAC
	MYC_REV	TTGGACGGACAGGATGTATGC

### Western blotting

For Western blotting 3 × 10^6^ cells were collected and lysed in RIPA extraction buffer (50 mM Tris–HCl (pH 7.4), 150 mM NaCl, 50 mM NaF, 2 mM EDTA, 1 Vol.-% NP-40, 0.5% w/v Natriumdeoxycholat, 0.1% w/v SDS, Protease and Phosphatase inhibitors). Thereafter, extracts were diluted in Laemmeli buffer and denaturated for 10 min at 95 °C. The samples were separated by 4–20% Mini-PROTEAN® TGX™ Precast Protein Gel (Bio-Rad Laboratories, Hercules, California, USA) using HiMark™ Pre-stained Protein Standard (Thermo Fisher Scientific, Waltham, Massachusetts, USA) for sizing and blotted onto a 0.45 μm PVDF transfer membrane (Thermo Fisher Scientific, Waltham, Massachusetts, USA). Blocking was done over-night using TBST containing 3% BSA. The membrane was then cut and incubated with either Anti-FAT1 antibody (ab190242, Abcam, Cambridge, UK) followed by second Anti Rabbit IgG HRP linked antibody or, as loading control, with β-Actin (D6A8) Rabbit mAb HRP conjugated antibody (both Cell Signaling Technology, Danvers, Massachusetts, USA). Blots were developed with an ECL development kit (Western Lightning Plus-ECL; PerkinElmer, Waltham, USA) and imaged with Image Reader LAS-4000 mini (FUJIFILM, Tokyo, Japan).

### FAT1 overexpression, knockout and knockdown

We established *FAT1* overexpression (OE), *FAT1* Knockdown (KD) and *FAT1* Knockout (KO) in the T-ALL cell line Jurkat. Transfection was done as electroporation using the Neon® Transfection System and Neon® Transfection System 10 µL Kit (both Thermo Fisher Scientific, Waltham, Massachusetts, USA). Transfection was performed following the Neon® protocol for Jurkat microporation and efficiency was checked by simultaneous transfection of a pGFPmax vector (Lonza, Basel, Switzerland) and analysis of the GFP signal measured by FACSCalibur (BD Pharmingen, Heidelberg, Germany). *FAT1* OE was implemented as previously described^56^ transfecting a plasmid encoding a truncated but functional FAT1 (pFAT1-Trunc; Supplementary Fig. 7b) as shown, characterized and kindly provided by Morris et al.^12^. We further selected transfected cells with G-418 (Sigma-Aldrich/Merck, Darmstadt, Germany) for the presence of the neomycin resistance. Baseline *FAT1* expression was surveilled by Western blotting and overexpression by additional RT-PCR with primers detecting both wildtype and truncated FAT1 (Supplementary Fig. 7d).

For *FAT1* KO, cells were transfected with sgRNA (Sequences: See below, Supplementary Fig. 7d, Integrated DNA Technologies, Inc., Leuven, Belgium) and Cas9 protein with NLS (PNA Bio, Thousand Oaks, California, USA) in a plasmid-free approach as previously described^57^. Transfected cells were seeded as single clones. KO success was confirmed by PCR with Terra™ PCR Direct Polymerase Kit according to the standard protocol (Takara Bio Europe, Saint-Germain-en-Laye, France) and lack of FAT1 protein in Western blotting at a predicted band size of 506 kDa. Interestingly, Western blot detected a second specific band for FAT1 with molecular weight between 71 and 117 kDa in accordance with the manufacture’s profile for FAT1 antibody detection in Jurkat and other cell lines.

To better reflect a biological continuum of expression we additionally used independent and specific *FAT1* siRNA (Hs_FAT_2 FlexiTube and Hs_FAT_3 FlexiTube siRNA, both directed against human FAT1, Qiagen, Venlo, The Netherlands). *FAT1* KD success was controlled by RT-PCR.

**Table:** 

Gene	sgRNA label	sgRNA (5′–3′)	Prepared sequence according to^57^
FAT1	FAT1_sg_01	TATCACTCTGACACCTGCCA	taatacgactcactataGGACACCTGCCAAGGAAGTCgttttagagctagaaatagc
	FAT1_sg_02	TCATAGTCAAAGTCCCAGCT	taatacgactcactataGGCCCAGCTAGGCTTCTGGAgttttagagctagaaatagc

### WST-1 proliferation and viability assay

Effects on cell proliferation by *FAT1* OE, KD and KO were examined using the WST-1 assay from Roche (Basel, Switzerland). Cells were seeded into 12-well plates in 100 µl of cell suspension in a concentration of 0.5 × 10^6^ cells/ml. After defined time points (24 h to 96 h, depending on experimental condition) 100 µl of WST-1 reagent was added in a 1:1 dilution with PBS (Biochrom, Berlin, Germany). Afterwards, the plates to be analyzed were incubated for two hours (37 °C, 5% CO2) to allow the tetrazolium salt WST-1 reaction to formazan. The formazan absorbance was measured with a Sunrise microplate absorbance reader (Tecan, Männedorf, Switzerland) at 450 nm. As reference wavelength, 620 nm was chosen.

### Statistics

All data are expressed as means ± SEM and a P value below 0.05 was considered to indicate statistically significant differences (* p < 0.05, ** p < 0.01, *** p < 0.001). Statistical analyses were performed using GraphPad Prism7 software (GraphPad Inc., San Diego, California, USA). Quantitative Data between two independent groups were compared with a two-tailed t-test expecting Student's t-distribution under the null hypothesis. Significance of gene coregulation with FAT1 expression in the Affymetrix dataset (GSE78132) was determined by ANOVA testing. Status of promotor methylation between two independent groups was also compared with a two-tailed t-test analyzing differences in median promotor methylation for each sample. To test correlation between the median promotor methylation score and FAT1-expression per sample, the Pearson correlation coefficient was calculated between FAT1pos and FAT1neg samples. Linear regression analysis was additionally performed.

### Ethics approval and consent to participate

According to the Declaration of Helsinki, all patients from the two independent cohorts gave written informed consent to participate in the GMALL studies, which were approved by an ethics board of the Johann Wolfgang von Goethe University, Frankfurt/Main in Germany.

## Supplementary Information

Supplementary Information 1.

Supplementary Tables.

Supplementary Figures.

## Data Availability

Gene expression data based on Affymetrix HG-U133 Plus 2.0 that were used and analyzed during the current study have been deposited in NCBI's Gene Expression Omnibus and are accessible through GEO Series accession number GSE78132^29^. RNA-seq and methylation data for FAT1 evaluated for correlation analyses have been deposited at the European Genome-phenome Archive (EGA), which is hosted by the EBI and the CRG, under accession number EGAS00001006025. Further information about EGA can be found on https://ega-archive.org "The European Genome-phenome Archive of human data consented for biomedical research" (http://www.nature.com/ng/journal/v47/n7/full/ng.3312.html). All other data analyzed during this study are included in this published article and its supplementary information files.

## Abbreviations

CCND1: Cyclin D1
ETP-ALL: Early T-cell precursor acute lymphoblastic leukemia
ΔFAT1: Truncated FAT1 isoform
FAT1: FAT atypical cadherin 1
FAT1neg: FAT1 negative
FAT1pos: FAT1 positive
FC: Fold change
GMALL: German Multicenter Study Group on Adult Acute Lymphoblastic Leukemia
GSEA: Gene set enrichment analyses
KD: Knockdown
KO: Knockout
LEF1: Lymphoid enhancer-binding factor 1
MYC: MYC proto-oncogene
OE: Overexpression
OS: Overall survival
p: P-value
PRKCA: Proteinkinase C-Alpha
RAG1: Recombination activating gene 1
RNA-Seq: RNA sequencing
RT-PCR: Real-Time PCR
siRNA: Small interfering RNA
T-ALL: T-cell acute lymphoblastic leukemia
TLX1: T-cell leukemia homeobox protein 1
