# Supplementary material for: FAT1 expression in T-cell acute lymphoblastic leukemia (T-ALL) modulates proliferation and WNT signaling
Source: Sci Rep. 2023 Jan 18;13:972. doi: 10.1038/s41598-023-27792-0 (PMC9849452; doi:10.1038/s41598-023-27792-0)
Supplement: Supplementary file 1 — Supplementary Information 1. [file 41598_2023_27792_MOESM1_ESM.pdf]

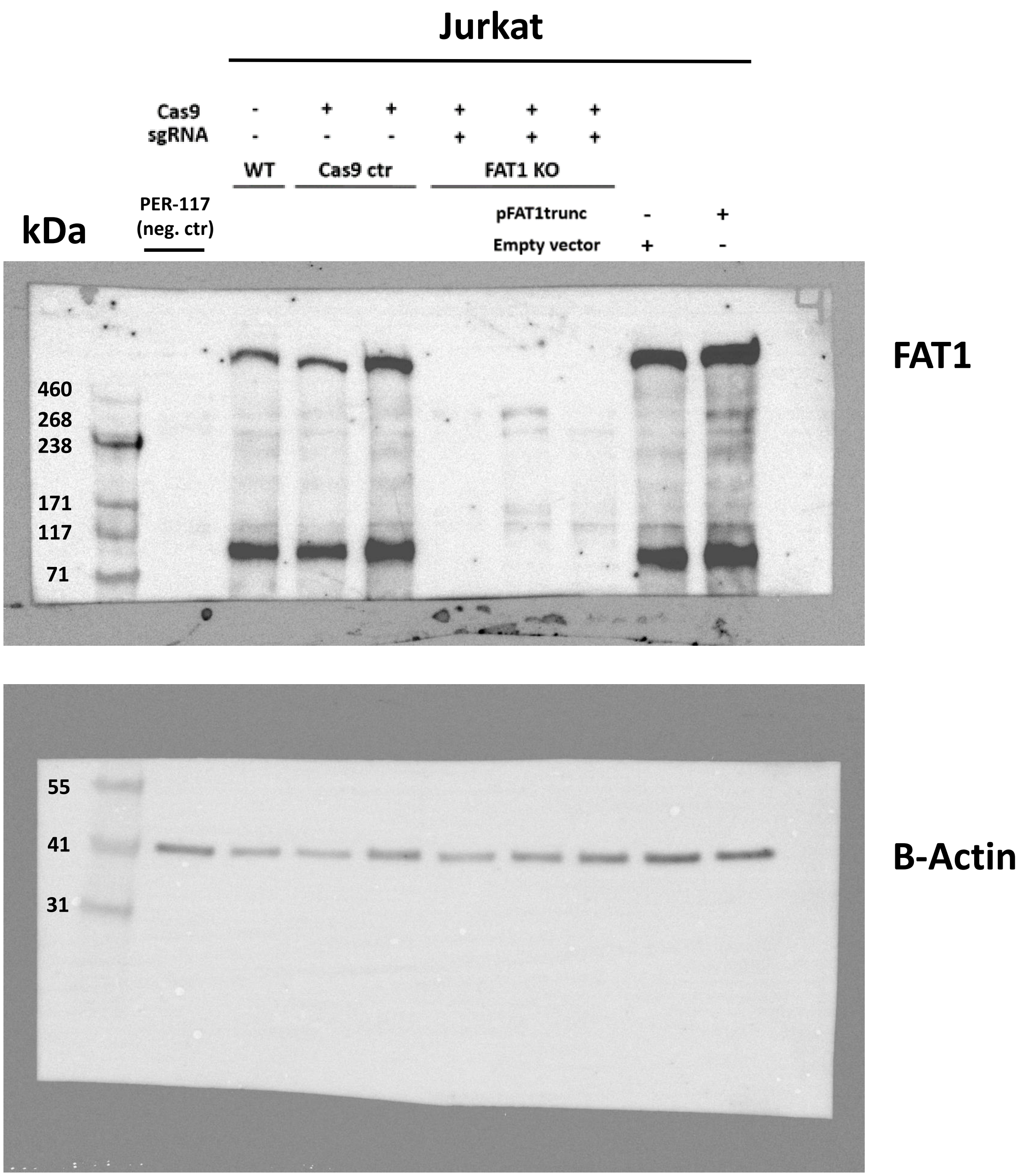

Supplementary information file: Full-length gel from Western blotting
corresponding to figure 4 (a) and (c)
