# Supplementary material for: FAT1 expression in T-cell acute lymphoblastic leukemia (T-ALL) modulates proliferation and WNT signaling
Source: Sci Rep. 2023 Jan 18;13:972. doi: 10.1038/s41598-023-27792-0 (PMC9849452; doi:10.1038/s41598-023-27792-0)
Supplement: Supplementary file 3 — Supplementary Figures. [file 41598_2023_27792_MOESM3_ESM.pdf]

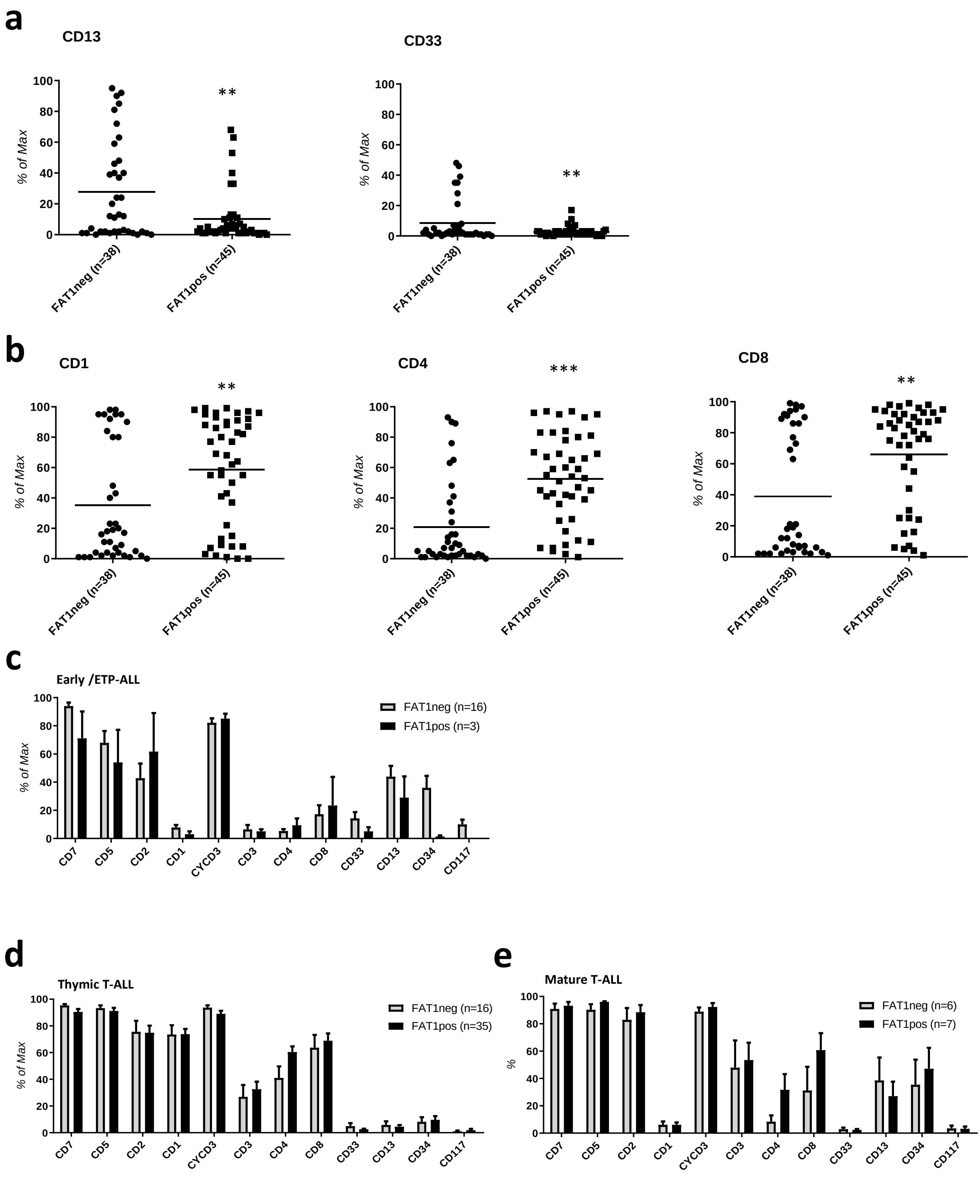

Supplementary figure 1: Flow cytometry based immunophenotyping for T-ALL samples according to *FAT1* expression. **(a-b)** FAT1 positive T-ALL patient samples show significantly higher levels of maturity markers CD1 (p=0.005), CD4 (p < 0.0001) and CD8 (p=0.001) compared to FAT1 negative patient samples, which show higher levels of CD13 (p=0.002) and CD33 (p=0.008). **(c-e)** Distribution of immunophenotyping markers according to FAT1 positivity in T-ALL subgroups Early/ETP-ALL, Thymic T-ALL and Mature T-ALL. CD4 and CD8 were higher for FAT1 positive patients in all subgroups alongside with lower levels of CD13 and CD33. (* p<0.05; ** p<0.01; *** p<0.001)

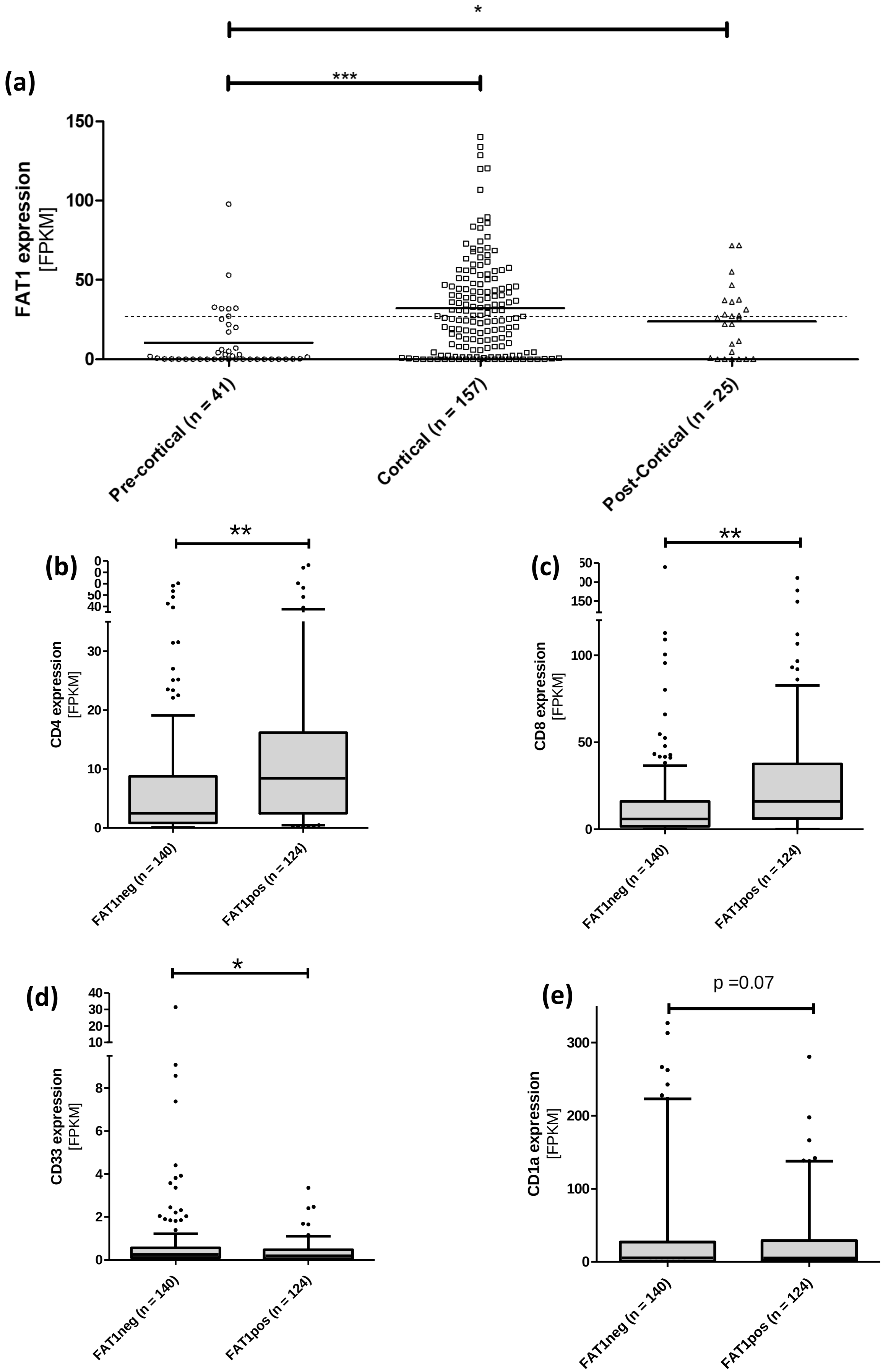

Supplementary figure 2: *FAT1* expression in T-ALL immune phenotypes validated in the Liu et al. dataset. **(a)** FAT1 expression is significantly higher in cortical (p<0.0001) and post-cortical-TALL (p=0.01) compared to pre-cortical T-ALL. 41 samples were not annotated for the immune phenotype. **(b-c)** Levels of T cell maturation markers CD4 (p=0.006) and CD8 (p=001) were significantly higher in FAT1pos (FPKM > 27; dotted line) compared to FAT1neg T-ALL samples. **(d)** Early myeloid marker CD33 (p=0.03) was lower in FAT1pos compared to FAT1pos T-ALL samples. **(e)** CD1a was not significantly different (p=0.07) between FAT1pos and FAT1neg. (* p<0.05; ** p<0.01; *** p<0.01)

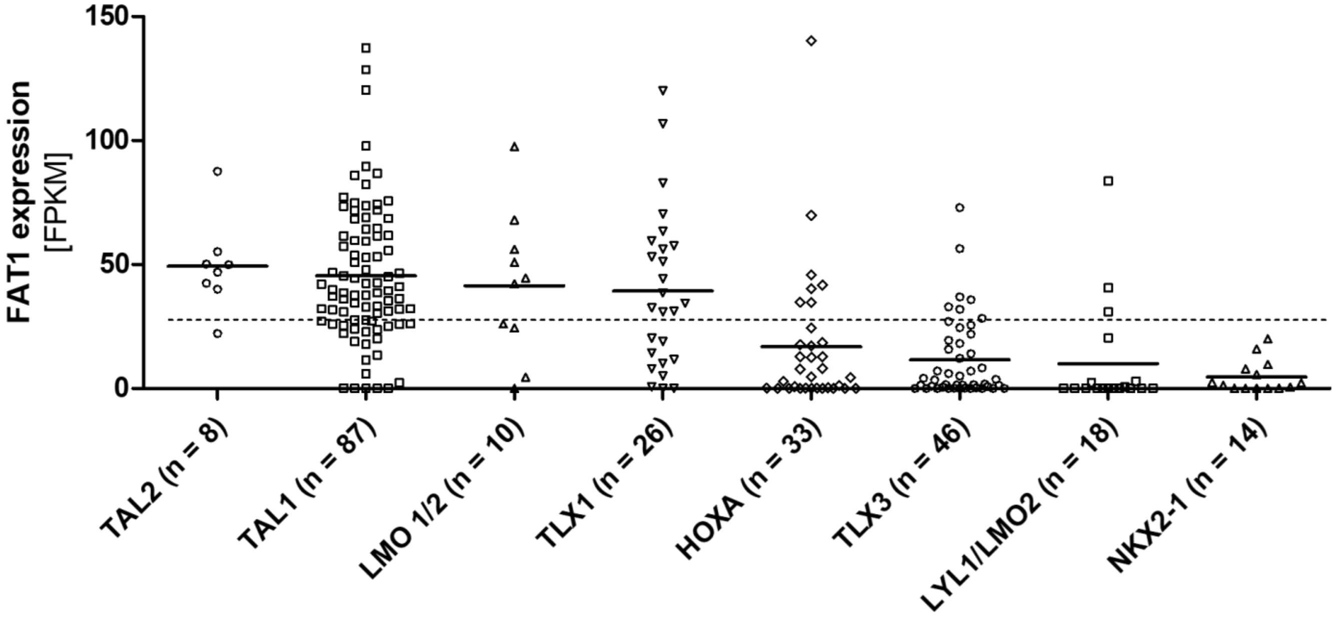

Supplementary figure 3: *FAT1* expression across molecular subtypes in T-ALL patients
In n = 264 samples from pediatric and young adult T-ALL samples of the Liu et al. (2013) cohort, FAT1 positivity (FPKM > 27; dotted line) was found in 47% of cases, mainly in TAL1/2, LMO1/2 and TLX1 subgroups. FAT1 was lower expressed (FPKM < 27) in all NKX2_1 and most HOXA, TLX3 and LYL1/LMO2 samples.

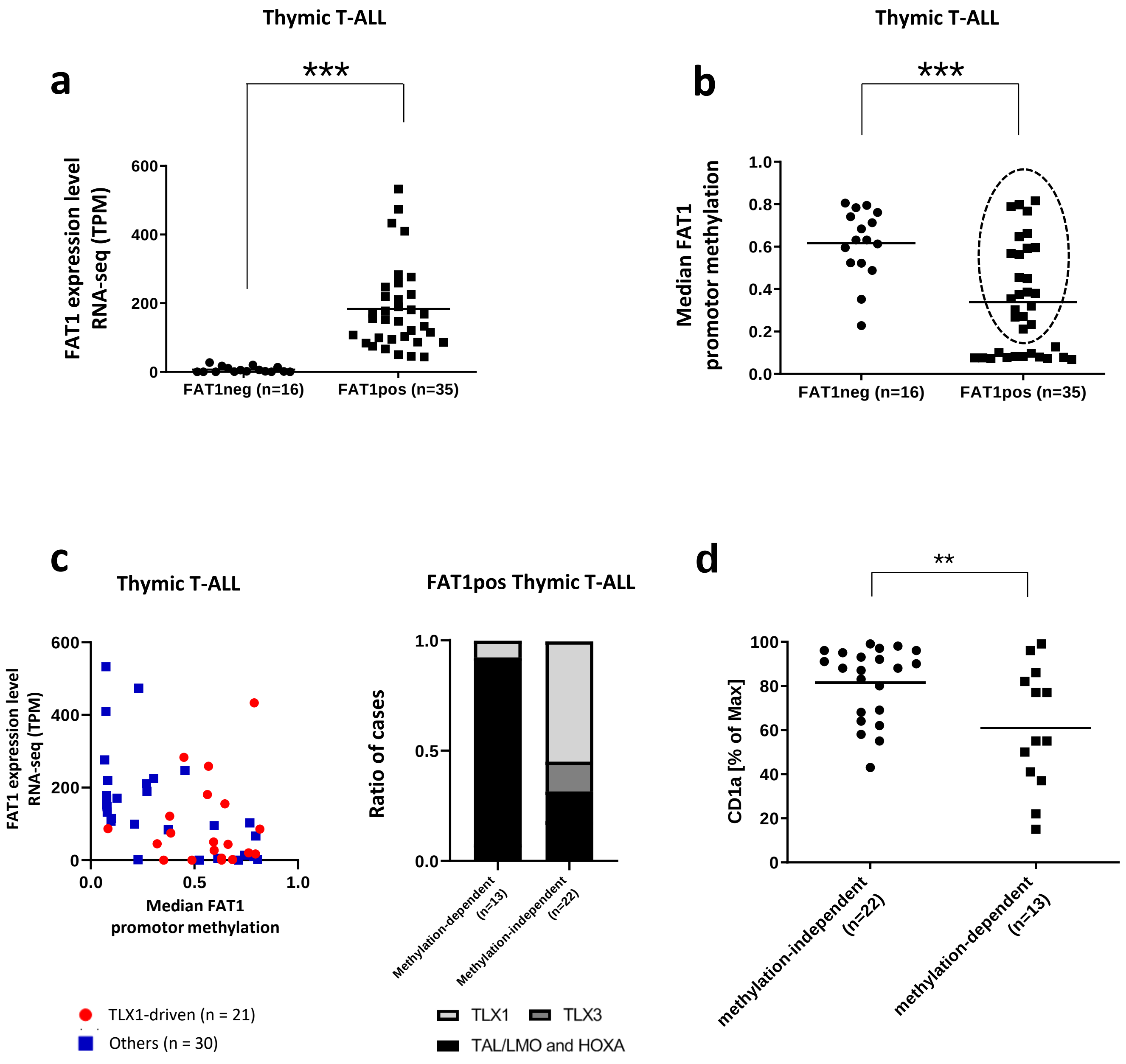

Supplementary figure 4: **Promotor methylation-independent *FAT1* expression in a Thymic T-ALL subgroup. (a-b)** *FAT1* expression in Thymic T-ALL (69% FAT1pos) shows a strong correlation with promotor hypomethylation. A subgroup of high expression and high promotor methylation (methylation-independent, marked by ellipse) could be identified. **(c)** The methylation independent *FAT1* expression subgroup mainly represents a TLX1-driven genetic subtype. **(d)** High levels of maturity marker CD1a in immunophenotyping represents TLX1 typical arrest of CD1+ CD4+ and CD8+ early cortical thymocytes. (** p<0.01; *** p<0.01)

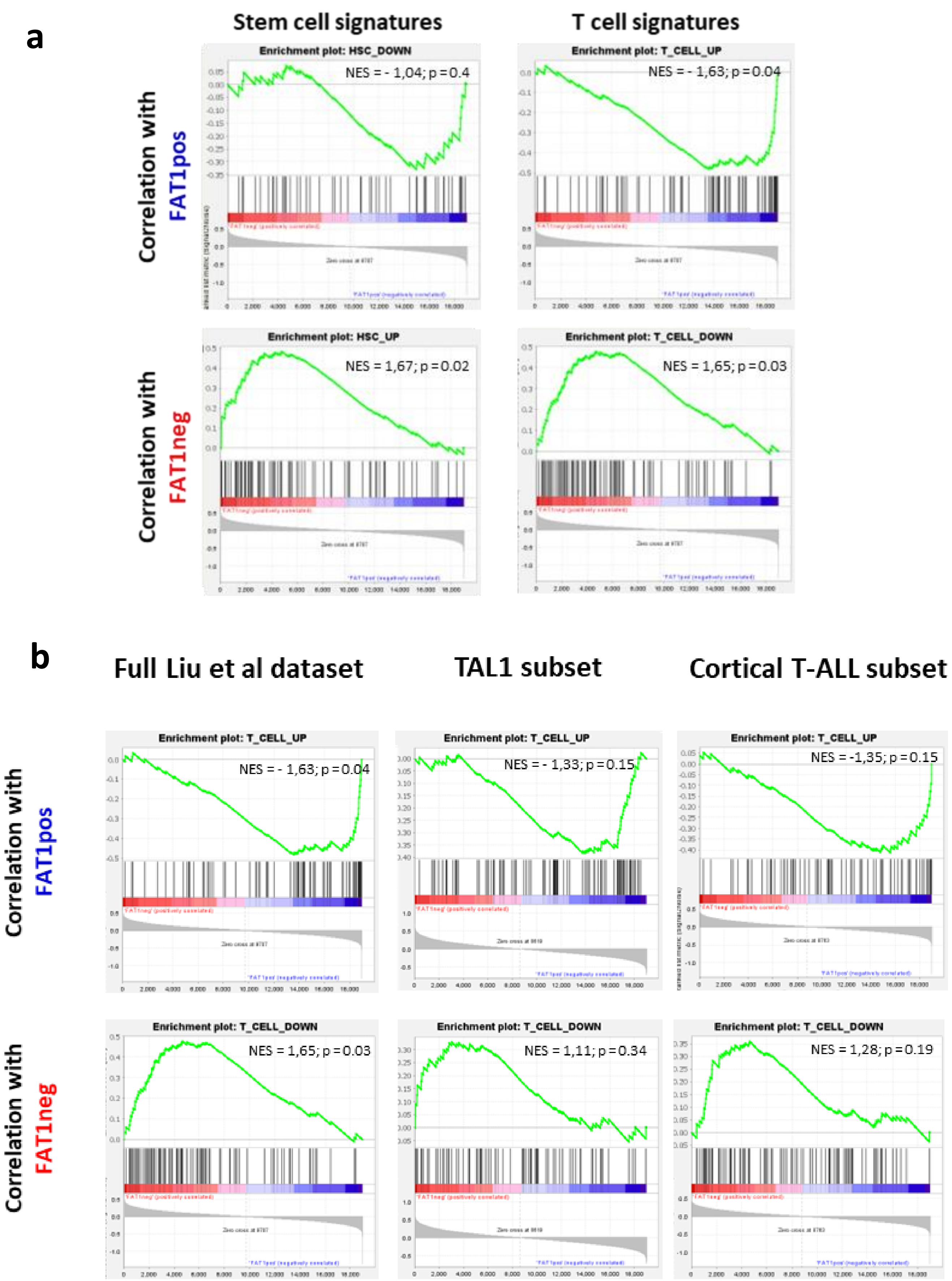

Supplementary figure 5: *FAT1* dependent expression of T cell maturation markers in the Liu et al. T-ALL dataset. (a) GSEA comparing FAT1pos with FAT1neg patients showed a strong correlation between FAT1 expression and T-ALL maturity with respect for mature T-cell and stem cell signatures**. (b)** The signature for genes upregulated in mature T-cells (upper series) was enriched for the FAT1pos phenotype in the full dataset and the most comprehensive subgroups. The signature for genes downregulated in mature T-cells (lower series) was enriched for the FAT1neg phenotype in the full dataset and the most comprehensive subgroups regarding molecular background (TAL1) and immune phenotype (Cortical T-ALL).

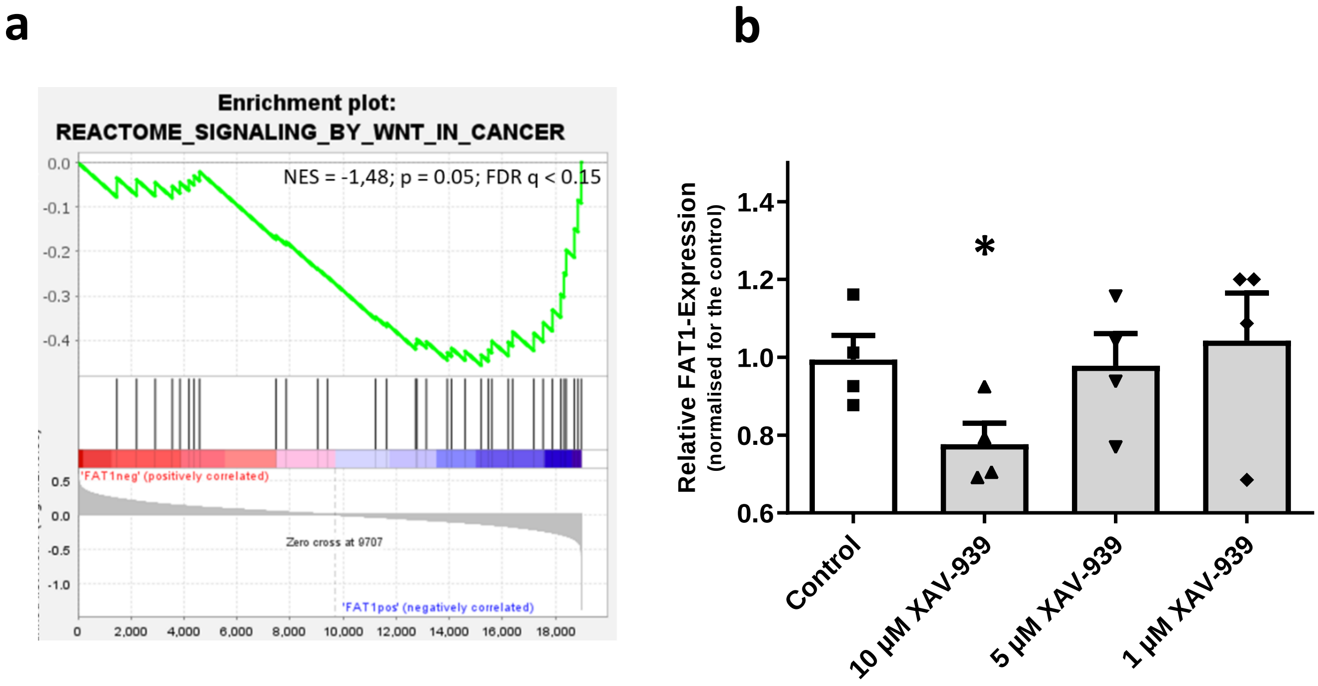

Supplementary figure 6: WNT pathway interaction with altered *FAT1* expression (a) In GSEA analysis from the Liu et al. dataset, the WNT pathway module was significantly enriched for the FAT1pos phenotype. **(b)** WNT pathway inhibitor XAV-939 inihibited FAT1 expression with dose dependency in Jurkat. Treatment with 10 µM XAV-939 for 24h resulted in a significant downregulation of FAT1 (p= 0.04)

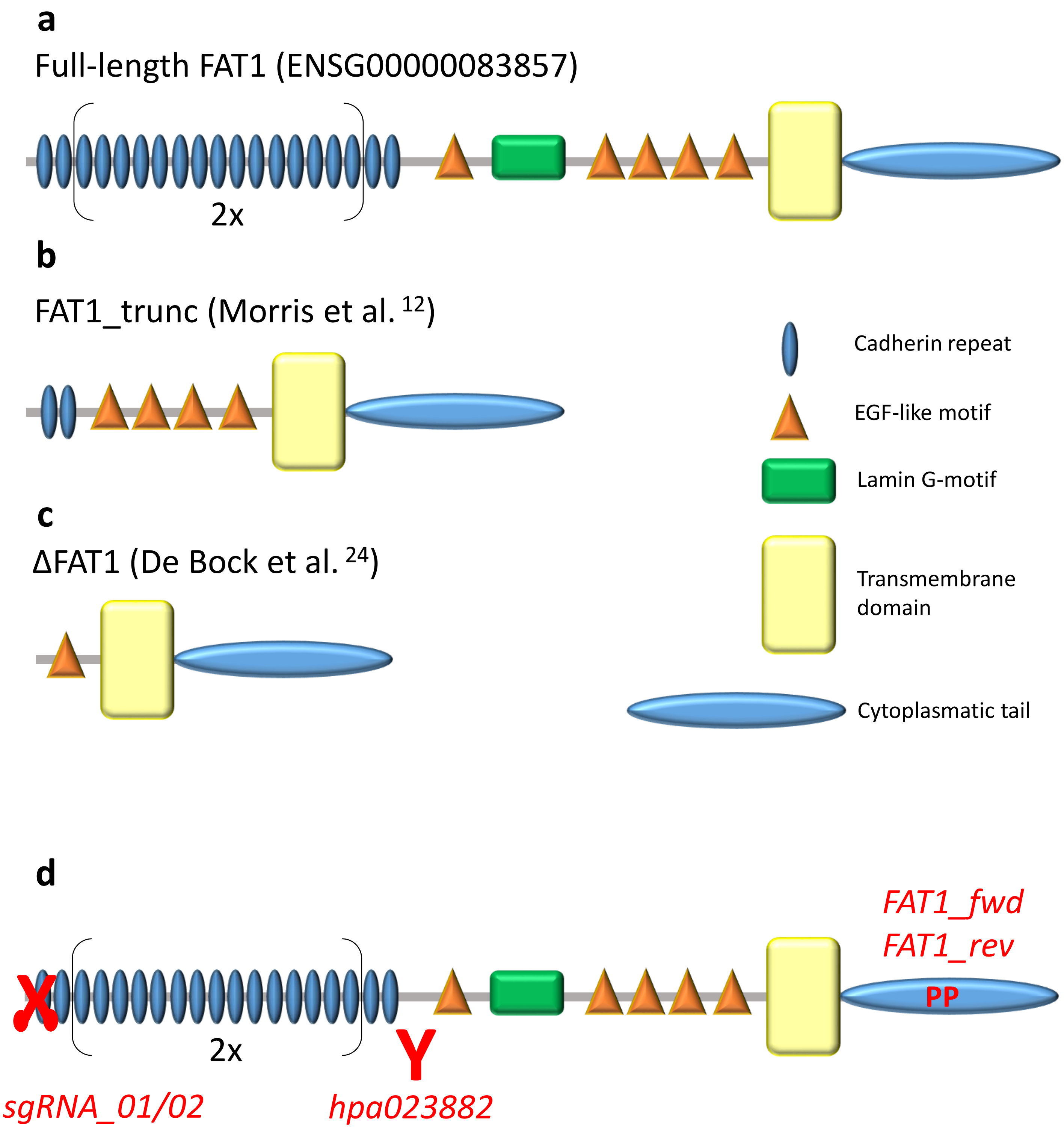

Supplementary figure 7: **Schematic overview of FAT1, its truncated variants and relevant sites**
(a) Schematic structure of full-length FAT1, consisting of 34 cadherin repeats, 5 EGF-like motifs, a Laminin G motif, the transmembrane domain and a cytoplasmatic tail. (b) Schematic structure of FAT1_trunc. (c) Schematic structure of ΔFAT1. (d) Schematic overview illustrating binding sites for the western blotting antibody used (hpa023882), the FAT1 primers (FAT1_fwd and FAT1_rev) and the sgRNAs targeting exon 1 (sgRNA_ 01/02).
